# A clinical case series of COVID-19-associated acute limb ischemia: real-world situation

**DOI:** 10.1186/s43044-021-00187-0

**Published:** 2021-06-30

**Authors:** Steven Philip Surya, Rony Marethianto Santoso

**Affiliations:** 1 Primaya Hopsital Tangerang, Jl. MH. Thamrin no.3, Kebon Nanas, Cikokol, Tangerang, Indonesia; 2 Department of Cardiology and Vascular Medicine, Primaya Hospital Tangerang, Tangerang, Indonesia

**Keywords:** COVID-19, Acute limb ischemia, Immunothrombus, Case report, COVID-19-associated coagulopathy

## Abstract

**Background:**

COVID-19 was a trending topic all year long in 2020. Currently, it is not only a problem for a pulmonologist since it could cause complications to many other organs, including the cardiovascular system. Recent acute COVID-19 infection state has been associated with hypercoagulation and causing microthrombi called immunothrombus. Acute limb ischemia is one of the rare complications but organ-threatening. Unfortunately, unlike coronary artery disease, there is no recent guideline for cardiologists to diagnose and manage acute limb ischemia in pandemic situations

**Case presentation:**

This case series presented two patients with acute limb injury (ALI)-complicating COVID-19, with chief complaints of pain at their lower extremity. The first patient was an 80-year-old woman who was just dismissed from the hospital due to COVID-19. The distal part of her toe was cyanosed, and her motoric and sensory functions were partially reduced. She was treated with oral drug therapy due to unwillingness to be hospitalized. Interestingly, she had recovered by using oral drug therapy. The second case was a 54-years-old female with several comorbidities such as obesity, type 2 diabetes mellitus, hypertension, dyslipidemia, and chronic obstructive pulmonary disease. She had cyanosed foot and weak arterial pulsation. Unfortunately, she passed away due to acute respiratory distress syndrome.

**Conclusion:**

Several internal and external factors cause ALI treatment to be more challenging in the pandemic COVID-19 situation. The diagnosis and management of ALI in COVID-19 patients may not fully comply with the current guideline and are likely to be affected by local hospital regulations. Clinical follow-up might be an essential feature in treating ALI in COVID-19 patients.

## Background

Coronavirus disease 2019 (COVID-19) has been a massive problem in our society and even more distressing for the medical community in 2020. World Health Organization (WHO) declared COVID-19 as a global disaster; moreover, the number of COVID-19 infected patients that we currently know might be an underestimation [1]. As one of the wide-spread infectious diseases, COVID-19 infection has a broad clinical spectrum from sub-clinical (only detected by laboratory test), mild symptoms (mostly outpatient), moderate symptoms (hospitalized non-intensive ward), severe symptoms (intensive ward), and unfortunately, death [2]. Initially, many clinicians believed COVID-19 was only associated with a respiratory infection; however, currently, we know that COVID-19 could affect other organ systems such as the cardiovascular system through angiotensin-converting enzyme 2 (ACE 2) and beyond [3, 4].

The correlation between COVID-19 and the cardiovascular system is widely known, but our understanding is much better in the heart organ than in blood vessels [5]. A case-control study between COVID-19 patient and control group showed that the case-group had a significantly higher number of lower-extremity arterial thrombus and increased risk of amputation [6]. A single-center study also discovered an escalation of acute limb ischemia cases in the first quarter of 2020 (COVID-19 pandemic) compared to the same period in 2019 [7]. Under normal circumstances, revascularization strategy, either endovascular or surgical techniques, might offer a better result in acute limb injury associated with arterial thrombus [8]. But in a real-world pandemic situation, the ideal situation might be harder to conduct due to internal and external reasons. Anticoagulant therapy might be the best decision that we can perform in the current pandemic situation [9]. This article addressed two patients with a chief complaint of pain in the lower extremity, later diagnosed with acute limb injury (ALI) in COVID-19 patients. The treatment described could be stood as a current recommendation.

## Case presentation

### Case 1

An 80-year-old woman presented with acute onset of devastating pain in the right lower extremity since a day ago. The severe aching sensation intensified within a few hours and reached its highest intensity (resting pain) in less than 24 h. There was no previous history of fever, dyspnea, myalgia, or even cough; however, she had just been discharged from the hospital due to confirmed COVID-19 pneumonia 1 week ago and was hospitalized for 15 days. Other than her old age, she only had stage I hypertension and dyslipidemia as her past medical history. She consumed candesartan 1 × 8 mg and multivitamins daily.

**Fig. 1:**
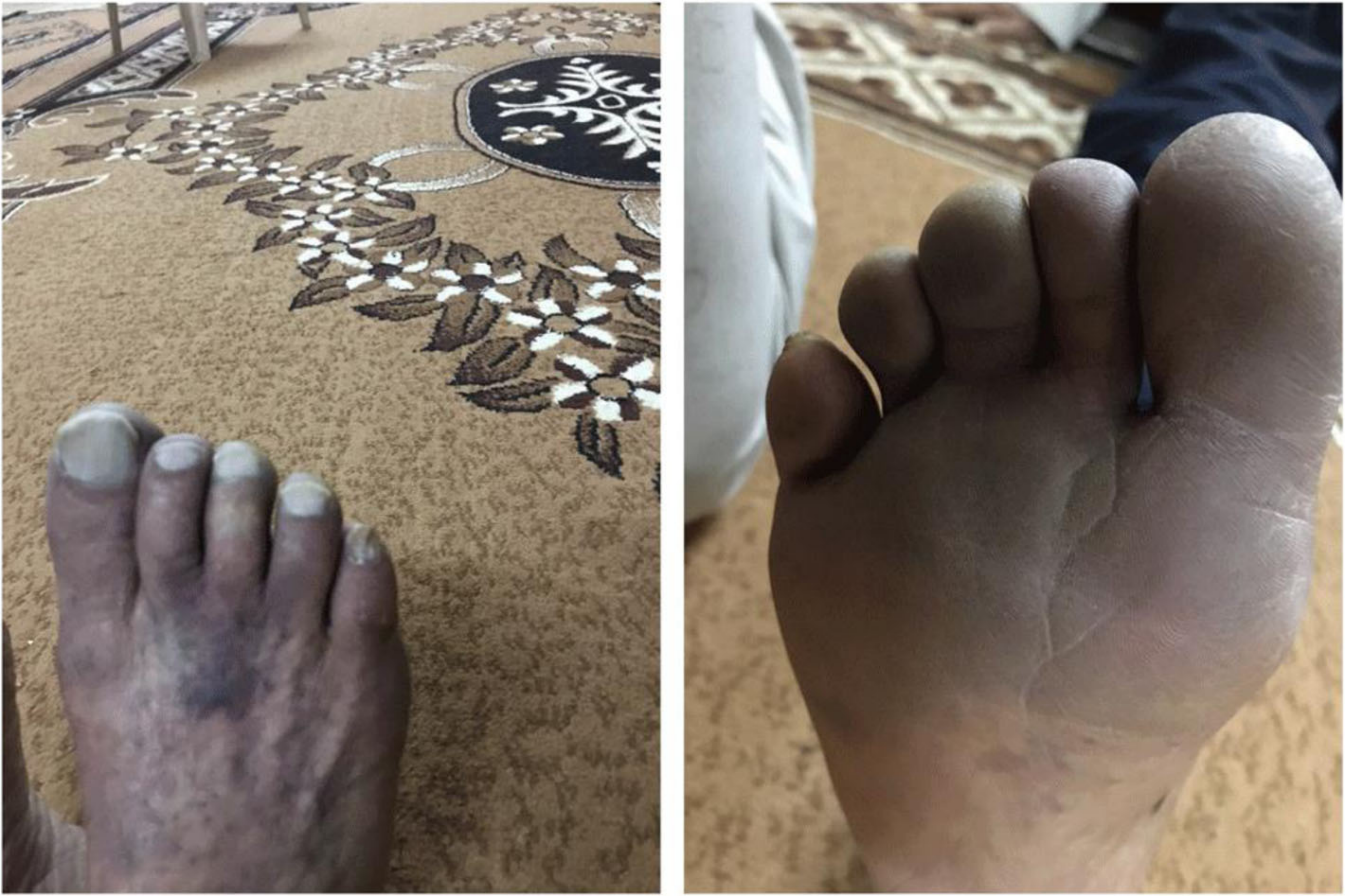
Right forefoot was cyanosed, and lower leg was cold, with reduced movement of toes

Her vital signs in the initial examination were unremarkable (blood pressure 130/80 mmHg, heart rate 100 beats per minute/bpm, respiratory rate 20 times per minute, oxygen saturation 95% in room air). On general examination, we found cyanosed and cold right forefoot (Fig. 1). She had a slight difficulty moving (motoric) her toe and a slight numb (sensory) in her toe. Lower extremity palpation revealed weak pulsation at the posterior and dorsal pedis artery locations, but her popliteal artery was still palpable.

**Fig. 2:**
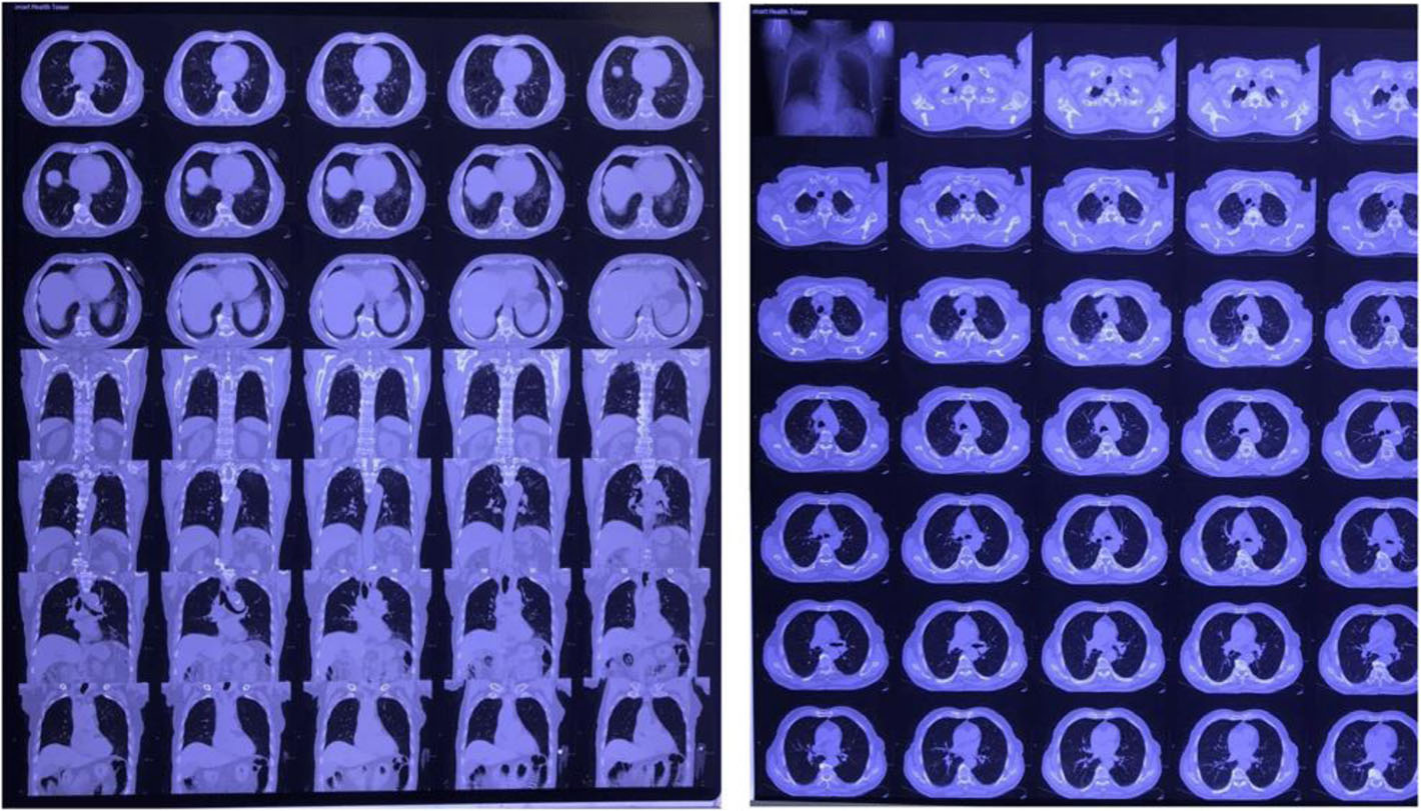
A CT scan of the patient’s chest showing multiple honeycomb appearance and ground-glass opacity (GGO)

Other than a mild decrease in hemoglobin (11.9 gr/dL) and mild leukocytosis, her routine blood examination was normal. The absolute lymphocyte count was 2200/uL. Her PCR test for COVID-19 was negative since last week, and her current immune-serology anti-IgM and IgG SARS-CoV-19 results were non-reactive. Chest X-ray showed normal cardio-thoracic-ratio/CTR and clear lung interstitial. However, Chest CT scan found multiple bilateral honeycomb appearances and ground-glass opacity (Fig. 2). Unfortunately, coagulation markers such as D-dimer PT/aPTT, CRP, and INR were not performed due to the patient’s refusal.

**Fig. 3:**
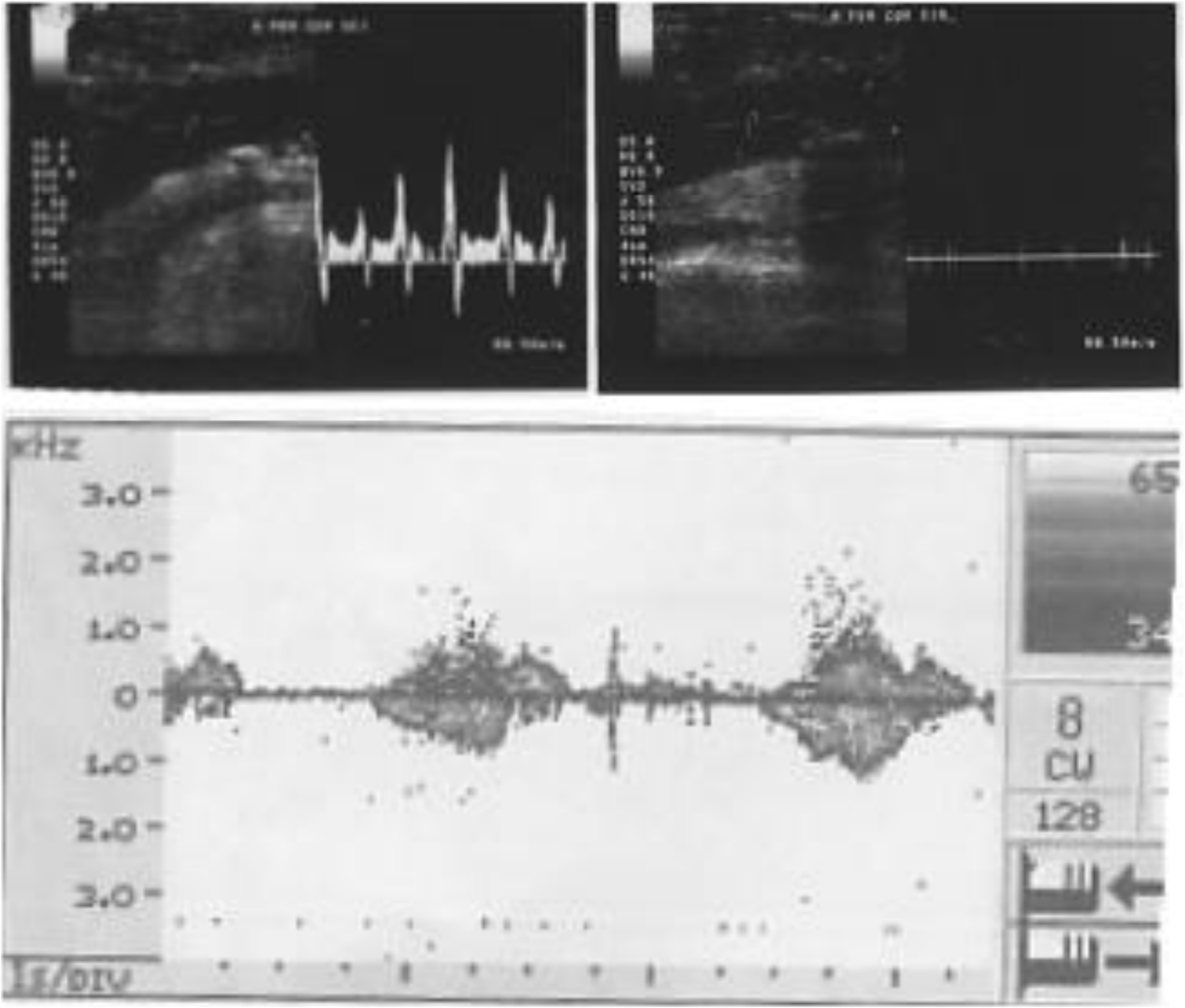
Normal flow velocity and spectrum in the common right femoral arteries (triphasic curve)

Doppler ultrasound examination showed normal flow velocity and spectrum in the common right femoral arteries (triphasic curve). Unfortunately, we found an occlusion and thrombus in the 1/3 proximal right popliteal artery with a minimum flow at the distal part of the posterior tibial and dorsalis pedis arteries. There were no clear signs of collaterals and no evidence of thrombus in the vein (deep vein thrombosis) (Fig. 3).

**Fig. 4:**
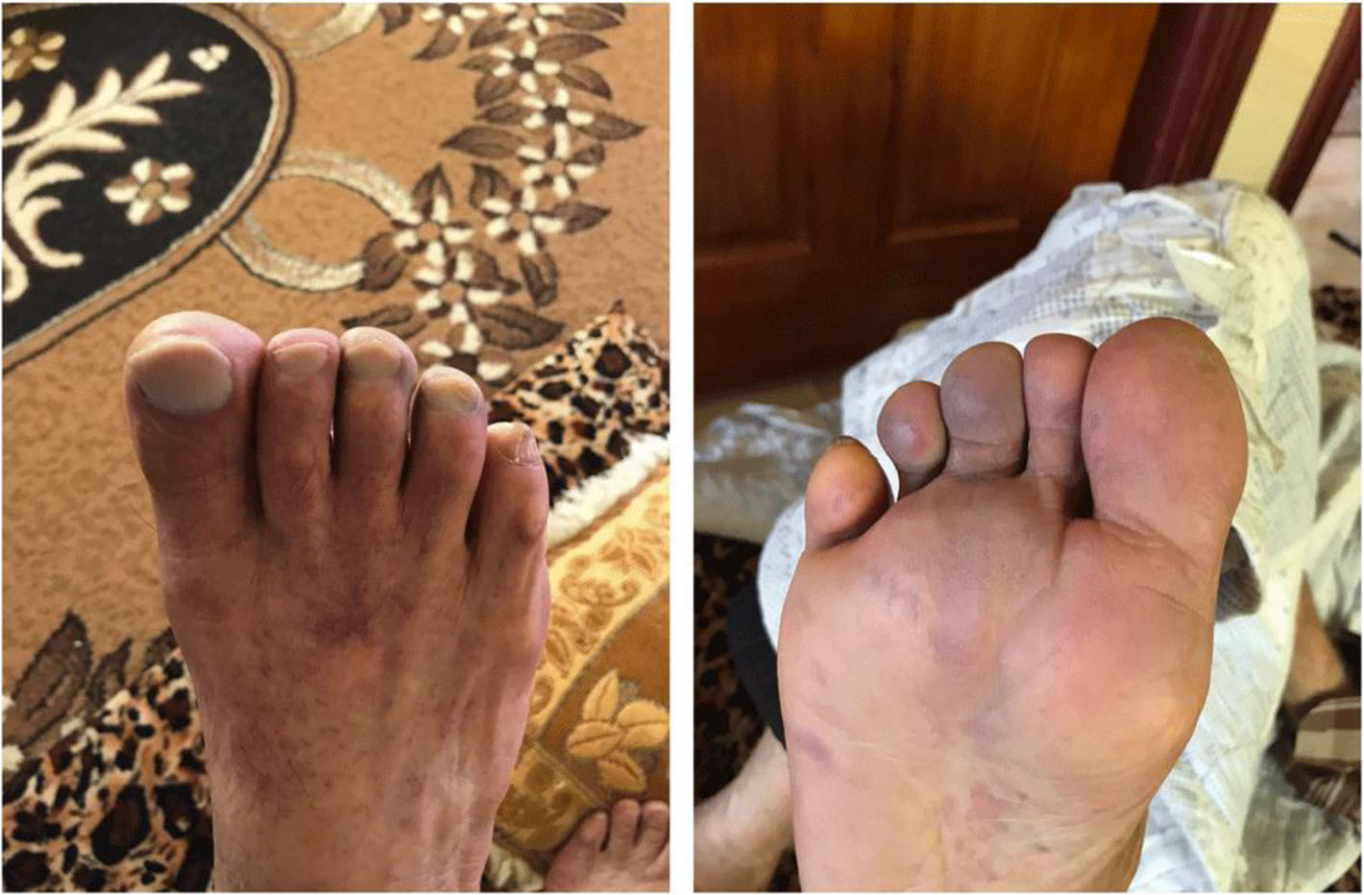
Four-week follow-up. Clinically improving, but the toe was still cyanosed

Based on our examination, the patient was diagnosed with acute limb injury classification IIa. Unfortunately, the patient refused to be hospitalized because she was just discharged from the hospital due to COVID-19. We prescribed the patient with aspilet 80 mg, atorvastatin 20 mg, cilostazol 2 × 100 mg, pentoxifylline 2 × 400 mg, candesartan 8 mg, enoxaparin 2 × 0.4 mg subcutaneously, and analgetic drug. In her 1-month follow-up, there was remarkable progress in the clinical appearance. The toe edges appeared to be well-perfused, with complete relief from pain and minimal sign of ischemia. Functionally, she could walk actively with no claudication (Fig. 4).

### Case 2

**Fig. 5:**
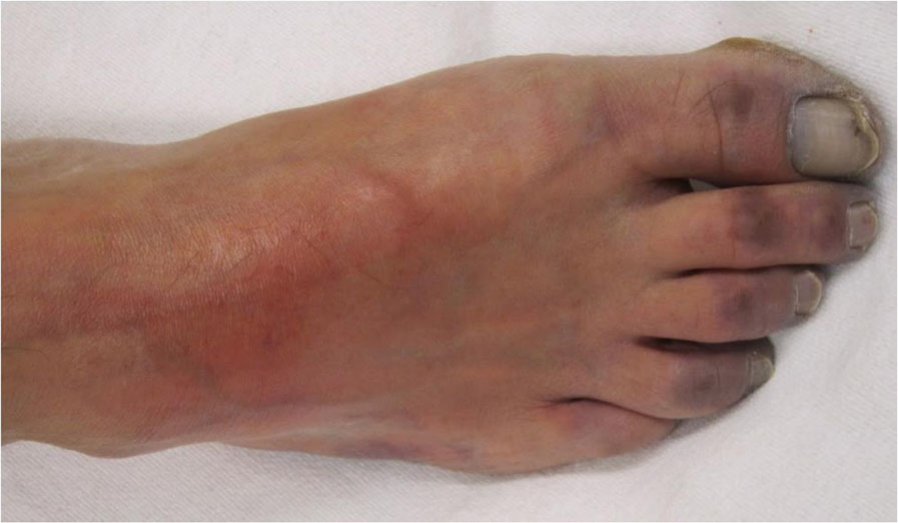
Weak pulse at posterior tibial and dorsalis pedis arteries. Cyanosed at toes

Another 54-year-old female presented to our hospital with a chief complaint of high-continuous fever for 1 week. She also had shortness of breath (dyspnea) in the last 7 days and had gradually worsened. She had a productive cough and high-intensity pain in her right leg. At the initial examination, she already had laboratory results with positive PCR result for COVID-19 and a raised D-dimer and fibrinogen. Our patient had many comorbidities such as obesity, type 2 diabetes mellitus, stage 2 hypertension, dyslipidemia, and chronic obstructive pulmonary disease (COPD). She routinely consumed amlodipine 10 mg, ramipril 5 mg, atorvastatin 20 mg, metformin 3 × 500 mg, and TSA capsule for COPD. Her initial vital signs suggested that her condition was unstable with tachypnea, tachycardia, elevated blood pressure 140/90 mmHg, and hyperpyrexia. Her peripheral oxygen saturation was 88-90% with non-rebreathing mask 10 L/min. Physical examination revealed no rhonchi and wheezing, yet chest X-ray indicated bilateral peripheral chest infiltrates. In further examinations of her lower extremities, we found tenderness of the right lower foot, weak pulsation at the posterior tibial and dorsalis pedis arteries. Distal toes were found to be cyanosed (Fig. 5).

Routine blood count (erythrocyte, leukocyte, hematocrit, hemoglobin, platelets) was showing within normal limits. Urea and creatinine serum level also indicated normal kidney function. Blood glucose level was also within the normal limit. Conversely, her absolute lymphocyte count was low, and neutrophil-lymphocytes-ratio (NLR) was > 3. Coagulation markers, including (D-dimer) and prothrombin time (PT), and activated partial thromboplastin time (aPTT) were dramatically increased. C-reactive protein, one of the markers believed to be associated with the severity of COVID-19 infection, was rapidly increased.

We performed a Doppler ultrasound examination, which was challenging in a pandemic situation. The Doppler ultrasound result showed normal flow velocity and spectrum in the common right femoral arteries (triphasic curve). Nevertheless, we discovered a significant occlusion and thrombus in the distal right popliteal artery, minimal flow in the distal posterior tibial and dorsalis pedis arteries. There was no clear sign of collaterals flow and no evidence of thrombus at vein (deep vein thrombosis).

The patient was hospitalized in the isolation ward with a diagnosis of COVID-19 and acute limb ischemia. She consumed aspilet 80 mg, atorvastatin 20 mg, cilostazol 2 × 100 mg, pentoxifylline 2 × 400 mg, amlodipine 10 mg, and ramipril 5 mg. She was also treated with unfractionated heparin (UFH) IV drip with a target control of 1.5×−2× of aPTT. The patient was prepared for further interventional-thrombolytic therapy; unfortunately, her condition worsened into acute respiratory distress syndrome, and our team decided to go on conservative treatment. She was intubated and ventilated. Two weeks later, her condition deteriorated, and she fell into septic shock. The patient had eventually passed away.

## Discussion

Acute limb ischemia (ALI) is a condition of sudden oxygen supply disturbance to the lower extremity. It is a medical emergency related to the viability of the limbs. International consensus prefers Doppler ultrasound (DUS) as the first-line imaging method in lower-extremity arterial diseases (LEAD) and additional imaging with either computed-tomography angiography (CTA) or magnetic resonance angiography (MRA) to determine the optimal revascularization management. However, it does not seem reasonable during the COVID-19 pandemic [10]. Symptoms of ALI might vary, including, not limited to, pain and deterioration of limb function. Limb’s viability must be assessed right after ALI was suspected [11]. Several factors that might affect the clinical presentation of ALI are location and duration of the arterial occlusion, the presence of collateral circulation, and the metabolic changes due to tissue ischemia [12].

A study from Dutch in early 2020 showed that around 31% of 184 critically ill COVID-19 patients admitted to Intensive Care Unit (ICU) had thrombotic complications (27% vein thrombotic and 3.7% arterial thrombotic) [13]. The condition known as COVID-associated coagulopathy (CAC), marked by elevated D-dimer level and the dysregulation of immune systems, is expected to play a central role in the complication of COVID-19 pneumonia patients [14]. In the acute infectious state, there is cross feedback between coagulation and the innate immune system, which develops thrombus called immunothrombus at the cellular level. Previous studies have shown that the pathogen-induced coagulation initially aimed to immobilize and kill the pathogen inside the clot [15]. Monocytes and neutrophils, the first responder of innate immune during an invasion, release an extracellular trap that promotes immunothrombosis right after exposure to pathogen-associated molecular patterns (PAMPs) and damage-associated molecular patterns (DAMPs). On the other hand, some types of leukocytes also contribute to fibrinolysis and thrombus formation [16]. Besides infection problems, COVID-19 patients also become susceptible to thromboembolism because of several factors, including prolonged immobilization, hypoxia, diffuse intravascular coagulation (DIC), and use of central vein catheter (CVC) [13].

After the virus gets to the respiratory epithelial cell through ACE2, the infected host cell will release DAMPs. Innate immune cells have pattern recognition receptors (PPR), which are essential in recognizing PAMPS and DAMPs, and eventually starting the inflammation cascade [17]. After the inflammatory pathway has been triggered, a pro-inflammatory cytokine, chemokine, and complement will be activated from the inactive form, which serves as the host response and resistance to the pathogen. At some point, over-recruitment of the pro-inflammatory cell, especially in the elderly, could cause damage to the host cell [18]. High pro-inflammatory cytokine, chemokine, and complement levels may trigger the thrombo-inflammatory process. This event has been clinically confirmed through chest computed tomography (Chest CT-Scan) or post-mortem autopsy examination [19, 20]. Viral pathogens, inflammatory cells, and mediators can induce tissue factor expression on monocytes and endothelial cell surfaces. The tissue factor acts as an activator of coagulation [21]. Moreover, inflammation causes imbalances between pro-coagulant and anti-coagulant states [22].

In case 1 and case 2, both patients were diagnosed with acute limb injury (ALI) with modified Rutherford classification grade IIA. In this situation, the limb organ viability was threatened but saveable if treated promptly [11]. The question is how fast and how ideal can we treat ALI patients in a pandemic situation? The American Heart Association (AHA) and American College of Cardiology (ACC) in Peripheral Artery Disease Guideline stated that prompt diagnosis and treatment must be performed to regain the skeletal muscle and other limbs organ’s function. Differentiation of a threatened and a nonviable limb is mandatory (the absence of arterial signal could indicate threatened limb, and the absence of both arterial and venous signals indicate irreversible condition) [11]. Ankle Brachial Index (ABI) can also be the simplest tool for predicting outcomes (ABI < 0.7 is considered critical) [12]. Despite other imaging modalities, such as DUS, MRA, and CTA, interventional angiography is the gold standard because it coincides with the necessary treatment [10–12, 23]. In the COVID-19 situation, imaging modalities might be challenging to perform due to hospital regulation, and patient transfers from the isolation ward might increase the risk of exposure to other patients. Additional cost for sterilization and protection gowns for the radiology staff might be costly.

According to the guideline, grade IIA ALI should undergo an emergency revascularization procedure within 6 h after the diagnosis has been made [13]. Revascularization strategy might consist of either interventional thrombolysis or surgical thromboembolectomy. Based on the underlying pathological process, unfractionated heparin/UFH (5000IU or 70-100 IU/kg) intravenously alongside analgesia with daily monitoring of activated clotting time (ACT) or activated partial thromboplastin time (APTT) are the commonly used initial therapy. However, the randomized control trial regarding the effectiveness of UFH and comparing UFH with other anticoagulants is still limited [12]. The limitation of this article is that we did not know the medication for COVID-19 therapy.

## Conclusion

The medical world has been under a catastrophic condition since pandemic COVID-19 in early 2020. Initially, COVID-19 was known to be associated with pneumonia. However, we learn later on that it causes concerning complications. As the number of COVID-19 patients increased, so did acute limb ischemia, especially in severely ill patients. ALI must be diagnosed and managed as soon as possible. Under normal circumstances, diagnosing and managing ALI might not be complicated; however, it is an entirely diverse situation in a pandemic. The challenging part might come from the patient due to refusal, hospital management related to isolation procedure, and sometimes from the clinician. Local hospital regulations might affect the diagnostic procedure and management of ALI during a pandemic. Multiple follow-ups with shorter intervals might be helpful while waiting for a recommendation for ALI in COVID-19 patients.

## Data Availability

Not applicable.

## Abbreviations

COVID-19: Coronavirus disease 2019
WHO: World Health Organization
ACE2: Angiotensin-converting enzyme 2
CTR: Cardio-thoracic-ratio
CRP: C-reactive protein
INR: International normalized ratio
PT: Prothrombin time
APTT: Activated partial thromboplastin clotting time
COPD: Chronic obstructive pulmonary disease
ALI: Acute limb injury
UFH: Unfractionated heparin
PAMPs: Pathogen-associated molecular patterns
DAMPs: Damage-associated molecular patterns
DIC: Diffuse intravascular coagulation
CVC: Central vein catheter
ABI: Ankle brachial index
MRA: Magnetic resonance angiography
CTA: Computed tomographic angiography
ACT: Activated clotting time
